# Comparison of the quadriceps-sparing and subvastus approaches versus the standard parapatellar approach in total knee arthroplasty: a meta-analysis of randomized controlled trials

**DOI:** 10.1186/s12891-015-0783-z

**Published:** 2015-10-31

**Authors:** Xiaochun Peng, Xianlong Zhang, Tao Cheng, Mengqi Cheng, Jiaxing Wang

**Affiliations:** Department of Orthopaedics, The Sixth Affiliated People’s Hospital, Shanghai Jiaotong University, Yishan Road, Shanghai, 200233 China

**Keywords:** Total knee arthroplasty, Standard parapatellar, Quadriceps-sparing, Subvastus, Meta-analysis, Randomized controlled trial

## Abstract

**Background:**

The quadriceps-sparing and subvastus approaches are two of the most commonly used minimally-invasive approaches in total knee arthroplasty (TKA). However, the conclusion among studies still remains controversial. The purpose of this meta-analysis was to compare the clinical efficacy of the subvastus and quadriceps-sparing approaches with the standard parapatellar approach in TKA.

**Methods:**

Randomized controlled trials (RCTs) comparing the quadriceps-sparing or subvastus approach with the standard parapatellar approach was identified in the databases of PubMed, the Cochrane library, EMBASE and Web of Science up to July 2014. Two authors extracted the following data: the basic characteristics of patients, the methodological quality and clinical outcomes from the included RCTs independently. RevMan 5.2.7 software was used for meta-analysis.

**Results:**

A total of 19 RCTs (1578 patients) were included for meta-analysis. The results suggested that the quadriceps-sparing approach showed better outcomes in knee society score (KSS) and visual analog score (VAS), but this approach required a longer operative time than the standard parapatellar approach. There were no differences in total complications, wound infection, deep vein thrombosis, blood loss and hospital stay between the quadriceps-sparing and standard approaches. The subvastus approach showed better outcomes in VAS, knee range of motion (ROM), straight leg raise and lateral retinacular release than the standard parapatellar approach. There were no differences in KSS, total complication, wound infection, deep vein thrombosis, blood loss and hospital stay between the quadriceps-sparing and standard approaches.

**Conclusions:**

The current evidence showed that, when compared with the standard parapatellar approach, the quadriceps-sparing approach was associated with better outcomes in KSS and VAS but required a longer operative time, and the subvastus approach was associated with better outcomes in VAS, ROM, straight leg raise and lateral retinacular release.

## Background

Total knee arthroplasty (TKA) has been one of the most successful operations for patients with end-stage knee diseases. As the standard approach, the medial parapatellar approach has been popularized for the excellent operative visualization. However, this approach requires the compromise of peripatellar blood supply and the quadriceps muscle, which might cause avascular necrosis and anterior knee pain [1].

In the last decade, the minimally invasive approaches such as mini-medial parapatellar, midvastus, subvastus and quadriceps-sparing approach, have provided promising advantages over the standard approach. The mini-medial parapatellar and midvastus approaches were less minimally invasive than the standard approach, but both disrupted quadriceps mechanism during surgery [2–4]. Compared with the above two approaches, the quadriceps-sparing and subvastus approaches were regarded as truly “anatomic” techniques in TKA because both avoided the disruption of the quadriceps tendon and the insertion of the vastus medialis in TKA [5–7]. Therefore, these two techniques also were described as the least minimally invasive approaches for TKA [8–10].

Theoretically, the quadriceps-sparing and subvastus approaches could offer better clinical outcomes for patients [11–15]. Previously, many studies have compared the clinical outcomes between the quadriceps-sparing or subvastus approach with the standard parapatellar approach. However, their conclusions among studies still remain conflicting. Some studies advocated the use of subvastus or quadriceps-sparing approach. They reported that these two approaches had significant advantages in knee society scores (KSS) [1, 16, 17], straight-leg raise [17–19], visual analogue score (VAS) [17] and range of motion (ROM) [16, 18, 20, 21]. However, other studies did not support this viewpoint. They found that the standard parapatellar approach provided less complications and better knee function than the subvastus or the quadriceps-sparing approaches [14, 19, 22–26].

To quantitatively compare the clinical efficacy and safety of the quadriceps-sparing and subvastus approaches to the standard parapatellar approach in TKA, we included all the published randomized controlled trials (RCTs) and conducted this meta-analysis.

## Methods

This meta-analysis was performed in accordance with the PRISMA (Preferred Reporting Items for Systematic Reviews and Meta-Analyses) guidelines [27].

### Inclusion criteria

The studies were included if they were randomized controlled trials (RCTs) comparing the subvastus or quadriceps-sparing approach with the standard parapatellar approach in TKA. Case report, cohort study, quasi-RCT and non-RCT were excluded in this study not considered for inclusion. The included participants should be adult patients who underwent the primary TKA. The extracted outcomes included: KSS and VAS, ROM, lateral retinacular release, straight leg raise, blood loss, operative time, hospital stay and postoperative complications (wound infection, deep vein thrombosis and total complications).

### Literature search

The databases of PubMed, the Cochrane library, EMBASE, Chinese Biomedical Database and ISI Web of Knowledge were searched for the relevant studies from January 1982 to July 2014. The following search strategies were used for literature search: #1, “Arthroplasty, Replacement, Knee” [Mesh]; #2, knee arthroplasty; #3. knee replacement, #4. medial parapatellar; #5. standard OR conventional approach; # 6. subvastus; #7. mini-subvastus; #8. quadriceps-sparing; #9. quad-sparing; #10. quadriceps sparing; #11. #1 OR #2 OR # 3 OR; # 12. #4 OR # 5; # 13. #6 OR #7 OR #8 OR # 9 OR #10; # 11 AND # 12 AND # 13. In addition, the lists of references and Google scholar were also searched for other potential RCTs.

### Data collection and quality assessment

Two authors independently screened the titles and abstracts. If the studies possibly met the inclusion criteria, the full text was retrieved for the final decision. Data extraction was completed by two blind authors. If insufficient data was reported, efforts were made to contact the authors for the additional information. The methodological quality was evaluated using the following items recommended by the Cochrane Collaboration [28]: randomization; allocation concealment; blinding of participants; blinding of outcome assessors; incomplete outcome data; selective reporting; and other bias. Each item was classified into “Yes”, “No”, or “Unclear”: “Yes” - low risk of bias, “No” - high risk of bias, “Unclear” - lack of information or unknown risk of bias. Any disagreement in assessments was resolved by discussing with a third author.

### Statistical analysis

The software of Review Manager 5.2.7 [28] was used to perform meta-analysis. Odds ratios (OR) and 95 % confidence interval (95 % CI) was calculated to test the overall effects for dichotomous outcomes, and mean difference (MD) and 95 % CI were used for continuous outcomes. Heterogeneity was tested using *I*^*2*^ statistic (*I*^*2*^ > 50 % indicating significant heterogeneity, and *I*^*2*^ ≤ 50 % indicating no significant heterogeneity) [29]. If significant heterogeneity (*I*^*2*^ > 50 %) was found in the meta-analysis, random-effect model was used, otherwise, using fix-effect model. Subgroup analysis was performed for outcomes with different time points.

## Results

**Fig. 1:**
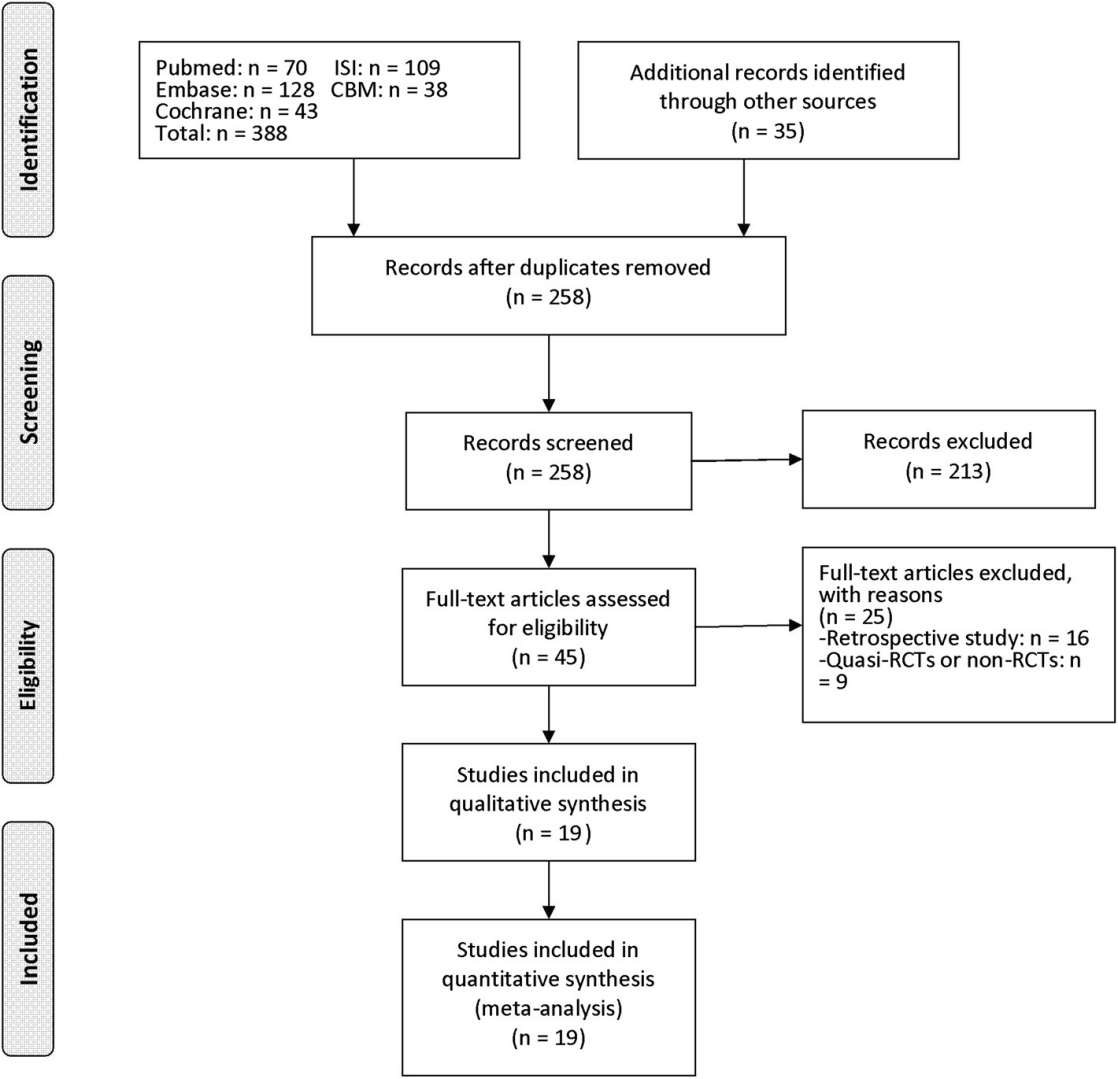
Flow chart of literature screening

Figure 1 showed the flow chart of literature screening. From the initial database search, a total of 423 citations were yielded. After removing 165 duplicates, 258 studies were reserved for abstract screening and full-text screening. Finally, nineteen RCTs [12, 14, 16, 18–26, 30–36] were considered to be eligible for meta-analysis. Of the included studies, nine [14, 17, 19, 22, 31–35] RCTs comparing the quadriceps-sparing approach with the standard parapatellar approach and ten [12, 16, 18, 20, 21, 23–26, 30] comparing the subvastus approach with the standard parapatellar approach were included.

### The characteristics and quality assessment of the included RCTs

The characteristics of the included RCTs were summarized in Table 1. A total of 1633 TKAs were performed in 1578 adult patients (male: 31.8 %; female: 68.2 %). The mean age ranged from 62.5–73.8, the mean BMI ranged from 24.6–30.97, and the follow-up duration ranged from 13 days to 3 years. The patients’ parameters (age, BMI, patient/TKA number, preoperative knee function) were reported similar between groups.

**Table 1:** Characteristics of included studies

Study-year	Country	Group	Patients (male/female)	Total TKA	Age	BMI	Follow-up	Results favoring
Chiang 2012	China	QS	30 (3/27)	38	69.7 ± 5.3	28.6 ± 3.8	2 years	MP
		SP	30 (3/27)	37	69.8 ± 5.4	29.6 ± 3.5		
Karpman 2009	America	QS	20 (8/12)	20	73 ± 7.4	28 ± 4.4	6 months	QS
		SP	19 (9/10)	19	73 ± 5.1	29 ± 4.6		
Kim 2007	Korea	QS	120 (27/93)	120	65.4 (43–88)	28.1 (19–36)	2 years	MP
		SP	120 (27/93)	120				
Lin 2009	China	QS	30 (3/27)	40	69.6 (57–78)	28.1 (20.1–36.9)	2 month	N.S
		SP	30 (3/27)	40	70.2 (56–82)	29.0 (20.1–36.9)		
Lin 2013	China	QS	35 (5/30)	35	67.7 ± 5	26.3 ± 2.5	2 years	QS
		SP	35 (5/30)	35	68.5 ± 5.5	25.9 ± 2.6		
Matsumoto 2011	Japan	QS	25 (0/25)	25	73.8 ± 1.7	Not reported	1 week	QS
		SP	25 (0/25)	25	73.7 ± 1.4	Not reported		
Shen 2007	China	QS	26 (−/−)	26	Not reported	Not reported	12 years	QS
		SP	33 (−/−)	33	Not reported	Not reported		
Tasker 2013	United Kingdom	QS	46 (17/29)	46	67.3 ± 8.4	Not reported	2 years	QS
		SP	46 (17/29)	46	68.2 ± 7.5	Not reported		
Xu 2013	China	QS	26 (7/19)	35	63.5 ± 8.7	25.2 ± 3.4	3 months	QS
		SP	29 (11/18)	35	64.2 ± 9.3	25.2 ± 2.3		
Roysam 2001	United Kingdom	SV	46 (25/21)	46	70.2	Not reported	3 months	SV
		SP	43 (22/21)	43	69.8	Not reported		
Weinhardt 2004	German	SV	26 (19/7)	26	69.7 ± 9.1	Not reported	13 days	N.S
		SP	26 (14/12)	26	73.7 ± 6.8	Not reported		
Bridgman 2009	United Kingdom	SV	116 (60/56)	116	70.1 ± 8.0	Not reported	1 year	SV
		SP	115 (59/56)	115	70.9 ± 8.1	Not reported		
Sastre 2009	Spain	SV	56 (not reported)	56	NR	Not reported	1 year	SV
		SP	48 (not reported)	48	Not reported	Not reported		
Pan 2010	China	SV	35 (11/24)	35	62.5 (54–70)	24.8 (19.5–28.6)	18 months	N.S
		SP	33 (9/24)	33	63.2 (50–75)	24.6 (19.4–28.2)		
Varela-Egocheaga	Spain	SV	50 (14/36)	50	68.02 ± 8.14	30.97 ± 5.25	3 years	SV
		SP	50 (13/37)	50	70.64 ± 7.88	30.62 ± 3.42		
Van Hemert 2010	Netherlands	SV	20 (6/14)	20	70.3 ± 11.8	29.2 ± 5.5	3 months	N.S
		SP	20 (7/13)	20	70.9 ± 7.1	30.3 ± 5.9		
Varnell 2011	Italy	SV	18 (11/7)	20	71 ± 6	30.96 ± 6.16	6 months	MP
		SP	15 (5/10)	18	70 ± 7	28.15 ± 4.2		
Wegrzyn 2013	USA	SV	18 (4/14)	18	71 ± 6	30.96 ± 6.16	2 months	N.S
		SP	18 (4/14)	18	70 ± 7	28.15 ± 4.2		
Jain 2013	India	SV	50 (12/38)	50	67 ± 8	30 ± 6	2 weeks	SV
		SP	50 (12/38)	50	64 ± 7	31 ± 4		

*BMI* body mass index, *SV* subvastus, *SP* standard parapatellar, *QS* quadriceps-sparing, *N.S* not significant

Regarding the methodological quality, all the included studies were randomized using various methods: eight (42.1 %) used the computer-generated random number and seven (36.8 %) used random number table. Allocation concealment was reported in 10 studies (52.6 %); the method of blind was used in 16 studies (84.2 %) (Table 2).

**Table 2:** Risk of bias in included studies

Study	Random Sequence Generation	Allocation concealment	Blinding of participants	Blinding of outcome assessment	Incomplete Outcome data	Selective reporting	Other bias
Kim 2007	Yes (Randomization table)	Unclear	Unclear	Yes	Yes	Unclear	Unclear
Shen 2007	Yes (Not reported)	Unclear	Unclear	Unclear	Yes	Unclear	Unclear
Karpman 2009	Yes (Computer)	Unclear	Yes	Yes	Yes	Unclear	Unclear
Lin 2009	Yes (Computer)	Yes (Sealed envelope)	Yes	Yes	Yes	Unclear	Unclear
Matsumoto 2011	Yes (Not reported)	Unclear	Yes	Unclear	Yes	Unclear	Unclear
Chiang 2012	Yes (computer)	Unclear	Yes	Yes	Yes	Yes	Unclear
Lin 2013	Yes (Randomization table)	Yes (Sealed envelope)	Unclear	Yes	Yes	Unclear	Unclear
Tasker 2013	Yes (Randomization table)	Yes (Sealed envelope)	Yes	Yes	Yes	Unclear	Unclear
Xu 2013	Yes (Randomization table)	Yes (Sealed envelope)	Unclear	Unclear	Yes	Unclear	Unclear
Roysam 2001	Yes (Random number table)	Yes (Sealed envelope)	Yes	Yes	Yes	Unclear	Unclear
Weinhardt 2004	Yes (Not reported)	Unclear	Unclear	Unclear	Yes	Unclear	Unclear
Bridgman 2009	Yes (Computer)	Yes (Telephone)	Yes	Yes	Yes	YES	Unclear
Sastre 2009	Yes (Random number table)	Yes (Sealed envelope)	Yes	Yes	No	Unclear	Unclear
Pan 2010	Yes (Computer)	Yes (Sealed envelope)	Yes	No	Yes	Unclear	Unclear
Varela-Egocheaga 2010	Yes (Random number table)	Unclear	Unclear	Unclear	Yes	Unclear	Unclear
Van Hemert 2010	Yes (Not reported)	Unclear	Yes	Yes	Yes	Unclear	Unclear
Bourke 2012	Yes (Computer)	Yes (Sealed envelope)	Yes	Yes	No	Unclear	Unclear
Wegrzyn 2013	Yes (Computer)	Unclear	Yes	Yes	Yes	Unclear	Unclear
Jain 2013	Yes (Computer)	Yes (Block schedule)	Yes	Unclear	Yes	Unclear	Unclear

### Results of meta-analysis

#### Quadriceps-sparing versus the standard parapatellar approach

Nine RCTs [14, 17, 19, 22, 31–35] comparing the quadriceps-sparing with the standard parapatellar approaches were included for meta-analysis (*n* = 725 patients with 775 TKAs) (Table 3).

**Table 3:** Meta-analysis of quadriceps-sparing (QS) versus standard parapatellar (SP) approach

Outcomes	Studies	No. of TKAs (QS/SP)		MD or OR (95 % CI); *p* value	Heterogeneity
KSS 4–6 weeks	3	101	108	−0.91 [−3.08, 1.25]; *p* = 0.41	*I* ^*2*^ = 0 %
KSS 3 months	2	138	138	2.88 [1.17, 4.60]; *p* = 0.001	*I* ^*2*^ = 0 %
KSS 2 year	2	155	155	1.75 [0.45, 3.06]; *p* = 0.008	*I* ^*2*^ = 0 %
VAS 1 weeks	4	124	129	−0.69 [−1.10, −0.29]; *p* < 0.05	*I* ^*2*^ = 32 %
VAS 4–6 weeks	3	104	110	0.14 [−0.29, 0.58]; *p* = 0.52	*I* ^*2*^ = 0 %
Total Complication	6	279	287	1.00 [0.21, 4.72]; *p* = 0.49	*I* ^*2*^ = 0 %
Wound infection	6	279	287	1.05 [0.39, 2.85]; *p* = 0.85	*I* ^*2*^ = 0 %
Deep vein thrombosis	6	279	287	0.67 [0.16, 2.92]; *p* = 0.30	*I* ^*2*^ = 0 %
ROM 1 week	2	64	70	5.79 [−6.26, 17.85]; *p* = 0.35	*I* ^*2*^ = 89 %
ROM 4–6 weeks	3	99	105	3.83 [−2.81, 10.46]; *p* = 0.26	*I* ^*2*^ = 84 %
ROM 3 months	2	146	153	4.37 [−6.41, 15.14]; *p* = 0.43	*I* ^*2*^ = 96 %
ROM 12 months	2	66	76	10.08 [−7.56, 27.72]; *p* = 0.26	*I* ^*2*^ = 96 %
ROM 24 months	4	233	235	−0.18 [−1.91, 1.56]; *p* = 0.84	*I* ^*2*^ = 0 %
Operative time	7	319	327	18.22 [9.92, 26.51]; *p* < 0.05	*I* ^*2*^ = 93 %
Blood loss	4	220	222	0.80 [−39.34, 40.93]; *p* = 0.97	*I* ^*2*^ = 0 %
Hospital stay	4	215	217	−0.68 [−1.48, 0.12]; *p* = 0.10	*I* ^*2*^ = 82 %

### Primary outcomes

Meta-analysis showed that, when compared with the standard approach, the quadriceps-sparing approach significantly improved KSS at postoperative 3 months (MD = 2.88, 95 % CI [1.17, 4.60], *P* = 0.001) and 2 years (MD = 1.75, 95 % CI [0.45, 3.06], *p* = 0.08), and decreased VAS at postoperative 1 week (MD = −0.69, 95 % CI [−1.10, −0.29], *P* < 0.05). There were no differences in KSS at postoperative 4–6 weeks (MD = −0.91, 95 % CI [−3.08, 1.25], *P* = 0.41) and VAS at postoperative 4–6 weeks (MD = 0.14, 95 % CI [−0.29, 0.58], *P* = 0.52) between both groups. No significant heterogeneity was found in the meta-analysis of KSS and VAS (*I*^*2*^ ≤ 50 %) (Table 3).

### Secondary outcomes

Meta-analysis showed that the quadriceps-sparing and the standard parapatellar approaches had similar results in total complication (MD = 1.00, 95 % CI [0.21, 4.72], *P* = 0.49), wound infection (MD = 1.21, 95 % CI [0.29, 5.05], *P* = 0.80), deep vein thrombosis (MD = 0.65, 95 % CI [0.13, 3.31], *P* = 0.60), ROM from 1 week (MD = 5.79, 95 % CI [−6.26, 17.85], *P* = 0.35 %)–24 months (MD = −0.18, 95 % CI [−1.91, 1.56], *P* = 0.84), blood loss (MD = −57.00, 95 % CI [−213.73, 99.73]) and hospital stay (MD = −2.00, 95 % CI [−3.19, −0.81], *P* = 0.10). However, the quadriceps-sparing approach significantly increased operative time when compared with the standard parapatellar approach (MD = 18.22, 95 % CI [9.92, 26.51], *P* < 0.05). The heterogeneity was significant in ROM, operative time and hospital stay (*I*^*2*^ > 50 %) (Table 3).

### Subvastus versus Medial parapatellar approach

Ten RCTs [12, 16, 18, 20, 21, 23–26, 30] comparing the subvastus with the standard parapatellar approach were included for meta-analysis (Table 4).

**Table 4:** Meta-analysis of subvastus (SV) versus standard parapatellar (SP) approach

Outcomes	Studies	TKAs (SV/SP)		MD or OR [95 % CI]; *p* value	Heterogeneity
KSS 4–6 weeks	2	128	127	−1.86 [−8.59, 4.88]; *p* = 0.59	*I* ^*2*^ = 66 %
KSS 3 months	4	196	195	1.03 [−10.28, 12.35]; *p* = 0.86	*I* ^*2*^ = 0 %
KSS 12 months	2	161	157	3.25 [−0.60, 7.10]; *p* = 0.10	*I* ^*2*^ = 0 %
VAS 1 weeks	2	132	133	−0.56 [−1.42, 0.29]; *p* = 0.19	*I* ^*2*^ = 98 %
VAS 4–6 weeks	3	182	175	−0.13 [−0.44, 0.19]; *p* = 0.44	*I* ^*2*^ = 85 %
VAS 3 months	3	182	177	−0.03 [−0.32, 0.27]; *p* = 0.87	*I* ^*2*^ = 84 %
VAS 6 months	2	165	159	−0.14 [−0.28, −0.01]; *p* = 0.04	*I* ^*2*^ = 0 %
Total Complication	6	329	315	0.81 [0.44, 1.49]; *p* = 0.49	*I* ^*2*^ = 0 %
Wound infection	6	242	233	1.11 [0.40, 3.08]; *p* = 0.85	*I* ^*2*^ = 0 %
Deep vein thrombosis	5	288	276	5.04 [0.24, 106.22]; *p* = 0.30	*I* ^*2*^ = 0 %
ROM 1 week	3	163	166	3.96 [3.20, 4.72]; *p* < 0.05	*I* ^*2*^ = 0 %
ROM 4–6 weeks	4	230	225	3.79 [−0.44, 8.03]; *p* = 0.08	*I* ^*2*^ = 68 %
ROM 3 months	4	230	225	3.24 [−0.90, 7.38]; *p* = 0.12	*I* ^*2*^ = 72 %
ROM 12 months	3	214	206	6.80 [0.94, 12.66]; *p* = 0.02	*I* ^*2*^ = 87 %
Straight leg raise	2	59	61	−2.77 [−4.07, −1.47]; *p* < 0.05	*I* ^*2*^ = 64 %
Operative time	2	46	46	0.11 [−10.37, 10.58]; *p* = 0.98	*I* ^*2*^ = 60 %
Lateral retinacular release	4	217	211	0.34 [0.14, 0.79]; *p* = 0.01	*I* ^*2*^ = 0 %
Blood loss	3	81	81	−100.76 [−223.42, 21.89]; *p* = 0.11	*I* ^*2*^ = 74 %

### Primary outcomes

Meta-analysis showed that the subvastus approach significantly reduced VAS score at postoperative 12 months (MD = −0.14, 95 % CI [−0.28, −0.01], *P* = 0.04) compared with the standard approach. There were no differences in KSS from postoperative 4 weeks (MD = −1.86, 95 % CI [−8.59, 4.88], *P* = 0.59)–12 months (MD = 3.25, 95 % CI [−0.60, 7.10]), and VAS from postoperative 1 week (MD = −0.56, 95 % CI [−1.42, 0.29], *P* = 0.19)–3 months (MD = −0.03, 95 % CI [−0.32, 0.27], *P* = 0.87) between the standard and subvastus groups (Table 3). Significant heterogeneity was found in KSS (4–6 weeks) and VAS (1 week–3 months) (*I*^*2*^ > 50 %).

### Secondary outcomes

Meta-analysis showed that the subvastus approach had significant advantages over the standard parapatellar approach in ROM at postoperative 1 week (MD = 3.96, 95 % CI [3.20, 4.72], *P* < 0.05) and 12 months (MD = 6.80, 95 % CI [0.94, 12.66], *P* < 0.05), straight leg raise (OR = −2.77, 95 % CI [−4.07, −1.47], *P* =0.02) and lateral retinacular release (OR = 0.34, 95 % CI [0.14, 0.79], *P* = 0.01). The two groups showed similar results in ROM at postoperative 4–6 weeks (MD = 3.79, 95 % CI [−0.44, 8.03], P = 0.08) and 3 months (MD = 3.24, 95 % CI [−0.90, 7.38], *P* = 0.12), total complication (MD = 0.81, 95 % CI [0.44, 1.49], *P* = 0.49), wound infection (MD = 1.11, 95 % CI [0.40, 3.08]) and blood loss (MD = −100.76, 95 % CI [−223.42, 21.89], *P* = 0.11) (Table 4).

## Discussion

Clinically, the quadriceps-sparing and subvastus approaches are very similar techniques, as both avoid the incision into the quadriceps tendon and the vastus medialis muscle during surgery. The quadriceps-sparing approach was first introduced by Tria et al. [7] from the minimally invasive unicondylar knee replacement. This technique used a more curvilinear medial incision without quadriceps damage and patella eversion. The advantage of the quadriceps-sparing approach was that, if needed, this technique can easily be extended or converted in the standard parapatellar approach [10]. However, critics indicated that this approach is not anatomically correct. Pagnano et al. [37] designed a magnetic resonance study in 200 cadaver specimens, and demonstrated that the vastus medialis obliquus was inserted to the midpole of the patella. Therefore, the quadriceps-sparing approach inevitably damaged the vastus medialis obliquus. The subvastus approach was first developed by Hofmann in 1991 [38]. It preserved the integrity of the extensor mechanism and minimized the injury to the patellar vascularity. Previous studies considered that the subvastus approach should be the true “quadriceps-sparing” approach in TKA [8, 18, 39].

For the subject concerning the superior approach for TKA, the conclusion was highly controversial. Among the included RCTs, five studies [17, 31, 32, 34, 35] favored the quadriceps-sparing approach, five [12, 16, 18, 20, 21] favored subvastus approach, four [14, 22, 30, 33] favored medial parapatellar approach, and others [19, 23–26] found no differences between groups. Therefore, we conducted a meta-analysis to quantitatively compare the clinical outcomes between the different approaches.

In our study, the most primary findings were that, the quadriceps-sparing approach had significant advantages in KSS and VAS over the standard approach, but had disadvantages in operative time. The subvastus approach provided better outcomes in VAS, ROM, straight leg raise and lateral retinacular release. There were no differences in other clinical outcomes when compared the quadriceps-sparing approach or subvastus with the standard group.

To date, there was no meta-analysis compared the quadriceps-sparing with the standard parapatellar approach in TKA. Totally, we included nine RCTs for meta-analysis. The results demonstrated that the quadriceps-sparing approach achieved better outcomes in KSS (postoperative 3 months and 2 years) and VAS (postoperative 1 week). This result supported the theory of minimally invasive technique. However, the level of this evidence was relatively weak due to the insufficient number of the included RCTs. In addition, our results also showed that the quadriceps-sparing group significantly increased operative time. The explanation was the quadriceps-sparing approach was technically more demanding. That required considerable efforts to obtain sufficient operative view during surgery [10].

Regarding the subvastus versus the standard parapatellar approach, ten RCTs were available for meta-analysis. Our meta-analysis showed that the subvastus approach had significant advantages over the standard approach in VAS (postoperative 6 months), ROM (postoperative 1 week and 12 months), straight leg raise and lateral retinacular release, and no disadvantages were found associated with the subvastus approach. Our conclusion was a little different with the published meta-analysis [1, 40]. Teng et al. [1] performed a meta-analysis including 8 RCTs and 1 quasi-RCTs, and concluded that the subvastus approach improved KSS score and decreased lateral retinacular release compared with the parapatellar approach. However, they found similar ROM in the two groups. The possible reason is that one quasi-RCT they included might bias the result of the meta-analysis. Additionally, despite surgical difficulty was high for the subvastus approach, the operative time showed no difference between the two groups. Our conclusion was in accordance with earlier studies [1, 23, 25, 40, 41] who also found no difference in operative time between both groups. The familiar exposure and new specific instrumentation contributed to the learning curve of subvastus technique.

### Strengths and Limitations of this study

Two earlier systematic review or meta-analysis [1, 40] had compared the clinical efficiency between the subvastus and the standard aprraoch in TKA. However, the authors included quasi-RCT, which reduced the level of the evidence. Additionally, the published meta-analysis only investigated the short-term outcomes. The strengthens of this study included that: (1) the results of our meta-analysis were based on RCTs, which provided high-level evidence for clinical practice; (2) our study first reported a meta-analysis comparing the quadriceps-sparing with the standard approach.

Several limitations should be noted in our study. (1). Although some outcomes were reported in the full text, data was not sufficiently provided to perform meta-analysis. (2) Although efforts were made to minimize the heterogeneity by conducting subgroup analysis, for example, using random-effect model and setting strict inclusion criteria, the heterogeneity among the included studies was still significant in several meta-analyses, which might decrease the reliability of the conclusion. Readers should be cautious for the results when heterogeneity existed. (3) Although all the included RCTs used randomization, some RCTs did not used allocation concealment and blinding to the patients and surgeons, which also might lead to high risks of selection and detection bias; besides, the most RCTs included were performed in single center with small samples, therefore, multi-center RCTs with large-samples are still lacking to verify our conclusion. (4) For a superior approach in TKA, it should include the following criteria: simple technique, sufficient visibility, less complication rates and improve clinical outcomes. Obviously, the quadriceps-sparing or subvastus approach did not involve all the criteria above. Therefore, TKA surgeons should get a balanced perspective for the two approaches.

## Conclusion

Based on the current evidence, our study finds that, in comparison with the standard parapatellar approach, the quadriceps-sparing approach showed better outcomes in KSS and VAS, and the subvastus approach shows better outcomes in VAS, ROM, straight leg raise and lateral retinacular release, but the quadriceps-sparing technique requires longer operative time.

## Abbreviations

TKA: Total knee arthroplasty
RCTs: Randomized controlled trials
KSS: Knee society scores
VAS: Visual analogue score
ROM: Range of motion
OR: Odds ratios
CI: Confidence interval
MD: Mean difference
BMI: Body mass index
SV: Subvastus
SP: Standard parapatellar
QS: Quadriceps-sparing
N.S: Not significant

## References

[1] Teng Y, Du W, Jiang J, Gao X, Pan S, Wang J (2012). Subvastus versus medial parapatellar approach in total knee arthroplasty: meta-analysis. Orthopedics.

[2] Heekin RD, Fokin AA (2014). Mini-midvastus versus mini-medial parapatellar approach for minimally invasive total knee arthroplasty: outcomes pendulum is at equilibrium. J Arthroplasty.

[3] Li XG, Tang TS, Qian ZL, Huang LX, Pan WM, Zhu RF (2010). Comparison of the mini-midvastus with the mini-medial parapatellar approach in primary TKA. Orthopedics.

[4] Zhang Z, Zhu W, Gu B, Zhu L, Chen C (2013). Mini-midvastus versus mini-medial parapatellar approach in total knee arthroplasty: a prospective, randomized study. Arch Orthop Trauma Surg.

[5] Niki Y, Mochizuki T, Momohara S, Saito S, Toyama Y, Matsumoto H (2009). Is minimally invasive surgery in total knee arthroplasty really minimally invasive surgery?. J Arthroplasty.

[6] Hofmann AA, Plaster RL, Murdock LE (1991). Subvastus (Southern) approach for primary total knee arthroplasty. Clin Orthop Relat Res.

[7] Tria AJ, Coon TM (2003). Minimal incision total knee arthroplasty: early experience. Clin Orthop Relat Res.

[8] Scuderi GR, Tenholder M, Capeci C (2004). Surgical approaches in mini-incision total knee arthroplasty. Clin Orthop Relat Res.

[9] Tenholder M, Clarke HD, Scuderi GR (2005). Minimal-incision total knee arthroplasty: the early clinical experience. Clin Orthop Relat Res.

[10] Aglietti P, Baldini A, Sensi L (2006). Quadriceps-sparing versus mini-subvastus approach in total knee arthroplasty. Clin Orthop Relat Res.

[11] Lin TC, Wang HK, Chen JW, Chiu CM, Chou HL, Chang CH (2013). Minimally invasive knee arthroplasty with the subvastus approach allows rapid rehabilitation: a prospective, biomechanical and observational study. J Phys Ther Sci.

[12] Jain S, Wasnik S, Mittal A, Hegde C (2013). Outcome of subvastus approach in elderly nonobese patients undergoing bilateral simultaneous total knee arthroplasty: A randomized controlled study. Indian J Orthop.

[13] Jackson G, Waldman BJ, Schaftel EA (2008). Complications following quadriceps-sparing total knee arthroplasty. Orthopedics.

[14] Kim YH, Kim JS, Kim DY (2007). Clinical outcome and rate of complications after primary total knee replacement performed with quadriceps-sparing or standard arthrotomy. J Bone Joint Surg Br.

[15] Chen AF, Alan RK, Redziniak DE, Tria AJ (2006). Quadriceps sparing total knee replacement. The initial experience with results at two to four years. J Bone Joint Surg Br.

[16] Varela-Egocheaga JR, Suarez-Suarez MA, Fernandez-Villan M, Gonzalez-Sastre V, Varela-Gomez JR, Rodriguez-Merchan C (2010). Minimally invasive subvastus approach: improving the results of total knee arthroplasty: a prospective, randomized trial. Clin Orthop Relat Res.

[17] Shen H, Zhang XL, Wang Q, Shao JJ, Jiang Y (2007). [Minimally invasive total knee arthroplasty through a quadriceps sparing approach: a comparative study]. Zhonghua Wai Ke Za Zhi.

[18] Roysam GS, Oakley MJ (2001). Subvastus approach for total knee arthroplasty: a prospective, randomized, and observer-blinded trial. J Arthroplasty.

[19] Lin WP, Lin J, Horng LC, Chang SM, Jiang CC (2009). Quadriceps-sparing, minimal-incision total knee arthroplasty: a comparative study. J Arthroplasty.

[20] Bridgman SA, Walley G, MacKenzie G, Clement D, Griffiths D, Maffulli N (2009). Sub-vastus approach is more effective than a medial parapatellar approach in primary total knee arthroplasty: a randomized controlled trial. Knee.

[21] Sastre S, Sanchez MD, Lozano L, Orient F, Fontg F, Nunez M (2009). Total knee arthroplasty: better short-term results after subvastus approach: a randomized, controlled study. Knee Surg Sports Traumatol Arthrosc.

[22] Chiang H, Lee CC, Lin WP, Jiang CC (2012). Comparison of quadriceps-sparing minimally invasive and medial parapatellar total knee arthroplasty: a 2-year follow-up study. J Formos Med Assoc.

[23] Weinhardt C, Barisic M, Bergmann EG, Heller KD (2004). Early results of subvastus versus medial parapatellar approach in primary total knee arthroplasty. Arch Orthop Trauma Surg.

[24] Pan WM, Li XG, Tang TS, Qian ZL, Zhang Q, Zhang CM (2010). Mini-subvastus versus a standard approach in total knee arthroplasty: a prospective, randomized, controlled study. J Int Med Res.

[25] Van Hemert WL, Senden R, Grimm B, van der Linde MJ, Lataster A, Heyligers IC (2010). Early functional outcome after subvastus or parapatellar approach in knee arthroplasty is comparable. Knee Surg Sports Traumatol Arthrosc.

[26] Wegrzyn J, Parratte S, Coleman-Wood K, Kaufman KR, Pagnano MW (2013). The John Insall award: no benefit of minimally invasive TKA on gait and strength outcomes: a randomized controlled trial. Clin Orthop Relat Res.

[27] Moher D, Liberati A, Tetzlaff J, Altman DG, Group P (2009). Preferred reporting items for systematic reviews and meta-analyses: the PRISMA statement. J Clin Epidemiol.

[28] Higgins. JPT, Green. S. Cochrane Handbook for Systematic Reviews of Interventions Version 5.1.0 [updated March 2011]. The Cochrane Collaboration. Available at: www.cochrane-handbookorg. Accessed 2 June 2014

[29] Higgins JP, Thompson SG (2002). Quantifying heterogeneity in a meta-analysis. Stat Med.

[30] Varnell MS, Bhowmik-Stoker M, McCamley J, Jacofsky MC, Campbell M, Jacofsky D (2011). Difference in stair negotiation ability based on TKA surgical approach. J Knee Surg.

[31] Xu J, Liu C, Zhou S, Lin Y (2013). Total knee arthroplasty:Comparison between quadriceps sparing approach and medial parapatellar approach. J Clin Rehabilit Tissue Engineering Res.

[32] Tasker A, Hassaballa M, Murray J, Lancaster S, Artz N, Harries W (2014). Minimally invasive total knee arthroplasty; a pragmatic randomised controlled trial reporting outcomes up to 2 year follow up. Knee.

[33] Lin SY, Chen CH, Fu YC, Huang PJ, Lu CC, Su JY (2013). Comparison of the clinical and radiological outcomes of three minimally invasive techniques for total knee replacement at two years. Bone Joint J.

[34] Matsumoto T, Muratsu H, Kubo S, Mizuno K, Kinoshita K, Ishida K (2011). Soft tissue balance measurement in minimal incision surgery compared to conventional total knee arthroplasty. Knee Surg Sports Traumatol Arthrosc.

[35] Karpman RR, Smith HL (2009). Comparison of the early results of minimally invasive vs standard approaches to total knee arthroplasty: a prospective, randomized study. J Arthroplasty.

[36] Shen H, Zhang XL, Wang Q, Shao JJ, Jiang Y (2007). Minimally invasive total knee arthroplasty through a quadriceps sparing approach: a comparative study. Zhonghua Wai Ke Za Zhi.

[37] Pagnano MW, Meneghini RM, Trousdale RT (2006). Anatomy of the extensor mechanism in reference to quadriceps-sparing TKA. Clin Orthop Relat Res.

[38] Hofmann AA, Plaster RL, Murdock LE (1991). Subvastus (Southern) approach for primary total knee arthroplasty. Clin Orthop Relat Res.

[39] Schroer WC, Diesfeld PJ, Reedy ME, LeMarr AR (2008). Mini-subvastus approach for total knee arthroplasty. J Arthroplasty.

[40] Hu X, Wang G, Pei F, Shen B, Yang J, Zhou Z (2012). A meta-analysis of the sub-vastus approach and medial parapatellar approach in total knee arthroplasty. Knee Surg Sports Traumatol Arthrosc.

[41] Bourke MG, Jull GA, Buttrum PJ, Fitzpatrick PL, Dalton PA, Russell TG. Comparing outcomes of medial parapatellar and subvastus approaches in total knee arthroplasty: a randomized controlled trial. J Arthroplasty. 2012;3:347–53. e341. 10.1016/j.arth.2011.06.005 21831580

